# SagE induces highly effective protective immunity against *Streptococcus iniae* mainly through an immunogenic domain in the extracellular region

**DOI:** 10.1186/1751-0147-55-78

**Published:** 2013-11-12

**Authors:** Yun Sun, Li Sun, Ming-qing Xing, Chun-sheng Liu, Yong-hua Hu

**Affiliations:** 1 Key Laboratory of Experimental Marine Biology, Institute of Oceanology, Chinese Academy of Sciences, 7 Nanhai Road, Qingdao 266071, China; 2 University of Chinese Academy of Sciences, Beijing 100049, China; 3 Department of Clinical Laboratory, Qingdao Municipal Hospital, Qingdao 266000, China; 4 Yellow Sea Fisheries Research Institute, Chinese Academy of Fishery Sciences, Qingdao 266071, China

**Keywords:** *Streptococcus iniae*, DNA vaccine, Immune response, Virulence

## Abstract

**Background:**

*Streptococcus iniae* is a Gram-positive bacterium and a severe pathogen of a wide range of farmed fish. *S. iniae* possesses a virulence-associated streptolysin S cluster composed of several components, one of which is SagE. SagE a transmembrane protein with one major extracellular region named ECR. This study aimed to develop a SagE-based DNA candidate vaccine against streptococcosis and examine the immunoprotective mechanism of the vaccine.

**Results:**

We constructed a DNA vaccine, pSagE, based on the *sagE* gene and examined its immunological property in a Japanese flounder (*Paralichthys olivaceus*) model. The results showed that at 7 days post-vaccination, expression of SagE at transcription and translation levels was detected in the tissues of the vaccinated fish. After challenge with *S. iniae* at one and two months post-vaccination, pSagE-vaccinated fish exhibited relative percent survival (RPS) of 95% and 88% respectively. Immunological analysis showed that (i) pSagE significantly upregulated the expression of a wide range of immune genes, (ii) pSagE induced the production of specific serum antibodies that bound whole-cell *S. iniae*, and (iii) treatment of *S. iniae* with pSagE-induced antibodies blocked bacterial invasion of host cells. To localize the immunoprotective domain of SagE, the ECR-expressing DNA vaccine pSagEECR was constructed. Immunization analysis showed that flounder vaccinated with pSagEECR exhibited a RPS of 68%, and that pSagEECR induced serum antibody production and immune gene expression in a manner similar to, though to lower magnitudes than, those induced by pSagE.

**Conclusions:**

We in this study developed a DNA vaccine, pSagE, which induces highly protective immunity against *S. iniae*. The protective effect of pSagE is probably due to its ability to elicit systemic immune response, in particular that of the humoral branch, which leads to production of specific serum antibodies that impair bacterial infection. These results add insights to the immunoprotective mechanism of fish DNA vaccine.

## Background

*Streptococcus iniae* is one of the common bacterial pathogens associated with disease outbreaks in farmed fish [1,2]. It was first isolated from Amazon freshwater dolphin in the 1970s and has since become one of the leading fish pathogens [3]. *S. iniae* has a broad host range and is known to affect at least 27 species of fish, which include a large number of economically important species such as rainbow trout, tilapia, sea bass, channel catfish, barramundi, and Japanese flounder [4-9]. In China, streptococcal outbreaks have been reported in farmed freshwater and marine fish, notably flounder, turbot, tilapia, and red drum [10-14]. The frequency and outcome of disease outbreak are influenced by culture and environmental factors, and stress conditions, such as intensive aquaculture operations and high temperature, can lead to heavy stock mortality [15-17].

Experimental *S. iniae* vaccines in the forms of subunit vaccines [10,18], DNA vaccines [19], and attenuated live vaccines [20-23] have been reported by a number of research groups. However, none of these vaccines have been commercialized. To date, the only licensed vaccines against *S. iniae* are bacterins consisting of inactivated whole-cell bacteria. In tilapia, it has been reported that killed bacterial cells combined with extracellular products produced effective protection [24]. Bacterins have been used to immunize farmed fish in Israel, Australia, Chile, and Spain [2,25,26]. However, the protectivity of inactivated vaccines proved to be limited [27,28].

In China, studies on *S. iniae* vaccines have begun only in recent years, and no licensed vaccines are available for aquaculture use. In Shandong Province of east China, *S. iniae* is recognized as a particularly severe pathogen for flounder and turbot, which are the principal economic fish species of the local area. In a previous study, we reported the construction of *S. iniae* DNA vaccines based on the *sagF*, *G*, and *I* genes of the streptolysin S cluster, which is known to be involved in the virulence of *S. iniae*[29-31]. We found that DNA vaccine plasmid expressing each of these genes induced effective protection. Since the streptolysin S locus is composed of nine genes, these observations led us to wonder whether other components of the streptolysin cluster also possess immunoprotective potential. To investigate this question, we in this study developed a DNA vaccine based on the *sagE* gene*,* another component of the streptolysin S cluster. We examined the immune response induced by SagE and its effect on bacterial infection. In addition, we also localized the main immunogenic region of SagE. Our results provide a useful vaccine candidate for the control of *S. iniae* and add insights to the protection mechanism of teleost DNA vaccines.

## Methods

### Sequence analysis

The amino acid sequence of SagE (GenBank accession no. AF465842.1) was analyzed using the BLAST program at the National Center for Biotechnology Information and the Expert Protein Analysis System. Subcellular localization was predicted with PSORTb version 3.0.2. Signal peptide search was performed with SignalP 3.0.

### Plasmid construction and preparation

The primers used in this study are listed in Table 1. To construct pSagE, which expresses His-tagged SagE, *sagE* was amplified by PCR with primers SagEF1 and SagER1. The PCR product was inserted into pCN3 [32] at the SmaI site. pCN3 is a plasmid derived from pCI-neo (Promega, USA), a mammalian expression vector, and contains the human cytomegalovirus immediate-early enhancer/promoter, which promotes constitutive expression of cloned DNA inserts in mammalian cells, and the late SV40 polyadenylation signal, which increases the steady-state level of RNA. pSagEECR, which expresses His-tagged ECR, was constructed in the same fashion with the primer pair ECRF1/ECRR1. Endotoxin-free plasmid DNA was prepared using EndoFree plasmid Kit (Tiangen, Beijing, China). The purity of the purified DNA was analyzed spectrophotometrically by measuring absorbance at A_260/280_ and A_260/230_. The integrity of the plasmid DNA was assessed by agarose gel electrophoresis.

**Table 1:** Primers used in this study

**Primer**	**Sequences (5′→ 3′)**^**a**^
CNF1	CTTGCGTTTCTGATAGGCACCTA
CNR1	TGCGGGCCTCTTCGCTATT
ECRF1	CCCGGGACCGCCATGCGTTGCTTTCAAAA (SmaI)
ECRR1	GCTCCCGGGTAAGATAGCAAACCAT (SmaI)
ECRF2	CCCGGGATGCGTTGCTTTCAAAA (SmaI)
SagEF1	CCCGGGACCACCATGATTTTTGGAAAAAGTAGTAATGGA (SmaI)
SagER1	GCCCGGGCCTTCTTACCTTTGACTGAT (SmaI)
SagEF2	ATGGAAAACTTCTCACAGGACTC
SagECR	GCTAACGCATTCAACCACAAA

^a^Underlined nucleotides are restriction sites of the enzymes indicated in the brackets at the ends.

### Fish

Japanese flounder (*Paralichthys olivaceus*) (average 11.2 g) were purchased from a local fish farm and acclimatized in the laboratory for two weeks before experimental manipulation. Fish were fed daily with commercial dry pellets and maintained at 20°C in aerated seawater. Before experiment, fish (5% of the stock) were randomly sampled for examination of bacterial recovery from blood, liver, kidney, and spleen as reported previously [33], and no bacteria were detected. ELISA analysis indicated that the randomly selected fish were negative of serum antibodies against *S. iniae*.

### Vaccination

All vaccine plasmids were diluted in PBS to 150 μg/ml. For vaccination with pSagE, flounder were divided randomly into three groups (*N* = 70) and injected intramuscularly (i.m.) with 100 μl pSagE, pCN3, or PBS. At one and two months post-vaccination (pv), 25 fish were taken from each group and challenged via intraperitoneal (i.p.) injection with 100 μl *S. iniae* SF1 [10] that had been cultured in Luria-Bertani broth (LB) medium at 28°C to OD_600_ 0.8 and resuspended in PBS to 10^7^ CFU/ml. Vaccination with pSagEECR was performed in the same fashion. For all vaccination trials, mortality was monitored for 20 days**,** and dying fish were randomly selected for examination of bacterial recovery from liver, kidney, and spleen by plate count as described previously [33]. Relative percent survival (RPS) was calculated as described previously [34]. All vaccination trials were repeated once, and the mean mortality and RPS were given in the results.

### Detection of plasmid DNA and vaccine gene expression

Muscle, kidney, liver, and spleen were taken from vaccinated fish at 7 days pv. For plasmid detection, DNA was extracted from the tissues with the TIANamp DNA Kit (Tiangen, Beijing, China) and used for PCR analysis with the primer pairs CNF1/SagER1 or CNF1/CNR1 (Table 1). To detect vaccine gene transcription, total RNA was extracted and used for reverse transcription-PCR (RT-PCR) as described previously [32] with α-tubulin as a reference [35]. Detection of vaccine protein by immunocolloidal gold electron microscopy was performed as reported previously [32].

### Purification of recombinant protein

To obtain recombinant ECR (rECR), the plasmid pECR, which expresses ECR linked to a protein tag (Trx-tag) derived from the backbone plasmid pET32a (Novagen, San Diego, USA), the coding sequence of ECR was amplified by PCR with primers ECRF2 and ECRR1 (Table 1), and the PCR product was inserted into pET32a at the EcoRV site. Recombinant ECR (rECR), which was used for ELISA assay, was purified with nickel-nitrilotriacetic acid agarose (QIAGEN, Valencia, USA) according to manufacturer’s instructions. The purified protein was dialyzed for 24 h against phosphate-buffered saline (PBS) and concentrated using Amicon Ultra Centrifugal Filter Devices (Millipore, Billerica, MA, USA). The protein was analyzed by sodium dodecyl sulfate-polyacrylamide gel electrophoresis (SDS-PAGE) and visualized after staining with Coomassie brilliant blue R-250 [see Additional file 1].

### Enzyme-linked immunosorbent assay (ELISA)

Sera were collected from the vaccinated fish at one and two months pv and diluted 20 times in PBS. Serum antibodies against rECR was determined by ELISA, which was performed as reported previously [36]. Briefly, 96-well ELISA plates (Sangon, Shanghai, China) were coated with 0.05% (w/v) poly-L-lysine in coating buffer (0.159% Na_2_CO_3_, 0.293% NaHCO_3_, pH 9.6) for 1 h, followed by washing the plates 3× with coating buffer. The plates were then coated with 100 μl/well purified recombinant protein dissolved in coating buffer (10 μg/ml) and incubated at 4°C for overnight. The plates were washed 3× with coating buffer and coated with 1% bovine serum album (BSA) at 22°C for 2 h, followed by washing 3× with PBST (0.1% Tween-20 in PBS). Diluted sera were added in triplicate to the wells of the plates. After incubation at 37°C for 2 h and washing with PBST, mouse anti-flounder IgM monoclonal antibody (Aquatic Diagnostic Ltd, Stirling, Scotland, UK) was added to the plates. The plates were incubated and washed as above. Horse-radish peroxidase-conjugated goat anti-mouse IgG (Bios, Beijing, China) was added to the plates. Color development was performed with the TMB Kit (Bios, Beijing, China). The plates were read at 450 nm with a Precision microplate reader (Molecular Devices, Canada).

### Quantitative real time RT-PCR (qRT-PCR) to examine immune gene expression

Spleen was taken from the vaccinated fish at 24 h post-challenge. Total RNA extraction was performed as described above. qRT-PCR was carried out using the SYBR ExScript qRT-PCR Kit (Takara, Dalian, China) as described previously with α-tubulin as an internal reference [37].

### Binding of vaccine-induced antibodies to bacterial cells

*S. iniae* SF1 was cultured as above and resuspended in PBS to 10^8^ CFU/ml. Fifty microliters of serum from pSagE- or pCN3-vaccinated fish was added to 0.5 ml *S. iniae* suspension, and the cells were incubated at 22 C for 1 h. The cells were collected by centrifugation, washed with PBS, and resuspended in 1 ml PBS. Mouse anti-flounder IgM monoclonal antibody (Aquatic Diagnostic, Scotland, UK) (1/100 dilution) was added to the cells. The cells were incubated, washed, and resuspended in PBS as above. Fluorescein isothiocyanate (FITC)-labeled goat anti-mouse IgG (Bios, Beijing, China) (1/1000 dilution) was added to the cells. The cells were incubated, washed, and resuspended in PBS as above. Two hundred microliters of cell suspension was dropped onto a glass slide and observed with a fluorescence microscope (Nikon E800, Japan).

### Effect of pSagE-induced antibodies on S. iniae infection

Flounder FG-9307 cell line was cultured at 22 C in 96-well cell culture plates with Eagle’s minimum essential medium (MEM) (GIBCO, Invitrogen, USA) as described previously [38]. For cellular infection, *S. iniae* SF1 in PBS (10^8^ CFU/ml) was mixed with serum from pSagE- or pCN3-vaccinated fish (1/10 dilution) at an equal volume. The mixture was incubated at room temperature for 0.5 h, and 10 μl of the mixture was added to each well of FG cells. The plates were incubated at 30 C for 4 h and washed three times with PBS. The cells were lysed with 1% Triton X-100, and 50 μl lysate was plated in triplicate on LB agar plates. After incubation at 30 C for 48 h, the colonies that appeared on the plates were counted.

### Statistical analysis

All statistical analyses were performed with the SPSS 17.0 package (SPSS Inc., Chicago, IL, USA). Chi-square test with Yates’ correction was used for mortality analysis, and analysis of variance (ANOVA) was used for all other analyses. In all cases, the significance level was defined as *P* < 0.05.

## Results

### Sequence characterization of SagE

SagE is composed of 220 residues and predicted to be a cytoplasmic membrane protein. It contains a signal peptide (residues 1 to 25), two membrane-spanning regions (residues 39 to 61 and 74 to 96), and one major extracellular region (residues 97 to 143), which, for convenience, was named ECR [see Additional file 2].

### Vaccine plasmid construction and expression of the vaccine gene in fish tissues following immunization

**Figure 1:**
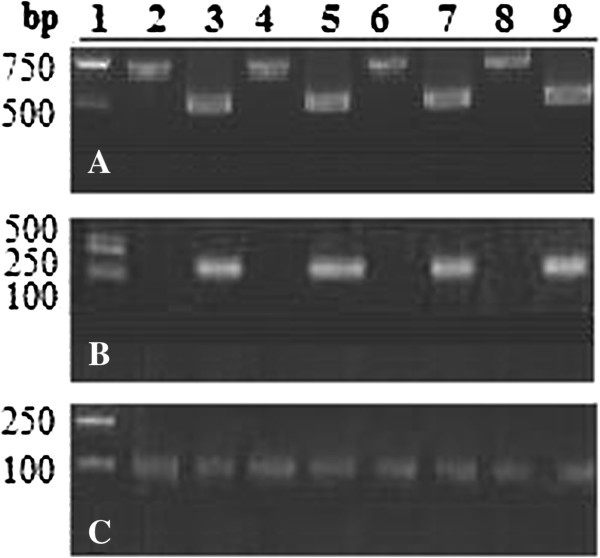
**Detection of vaccine plasmids (A) and expression of the vaccine-encoding genes (B and C) in fish tissues. (A)** Flounder were vaccinated with pSagE (lanes 3, 5, 7, and 9) or pCN3 (lanes 2, 4, 6, and 8). At 7 days post-vaccination, DNA and RNA were extracted from muscle (lanes 2 and 3), spleen (lanes 4 and 5), liver (lanes 6 and 7), and kidney (lanes 8 and 9) and used for PCR **(A)** and RT-PCR **(B and C)** analysis. PCR was performed using primers specific to pSagE or pCN3. RT-PCR was performed using primers specific to *sagE***(B)** or α-tubulin (internal reference) **(C)**. Lane 1, DNA markers.

**Figure 2:**
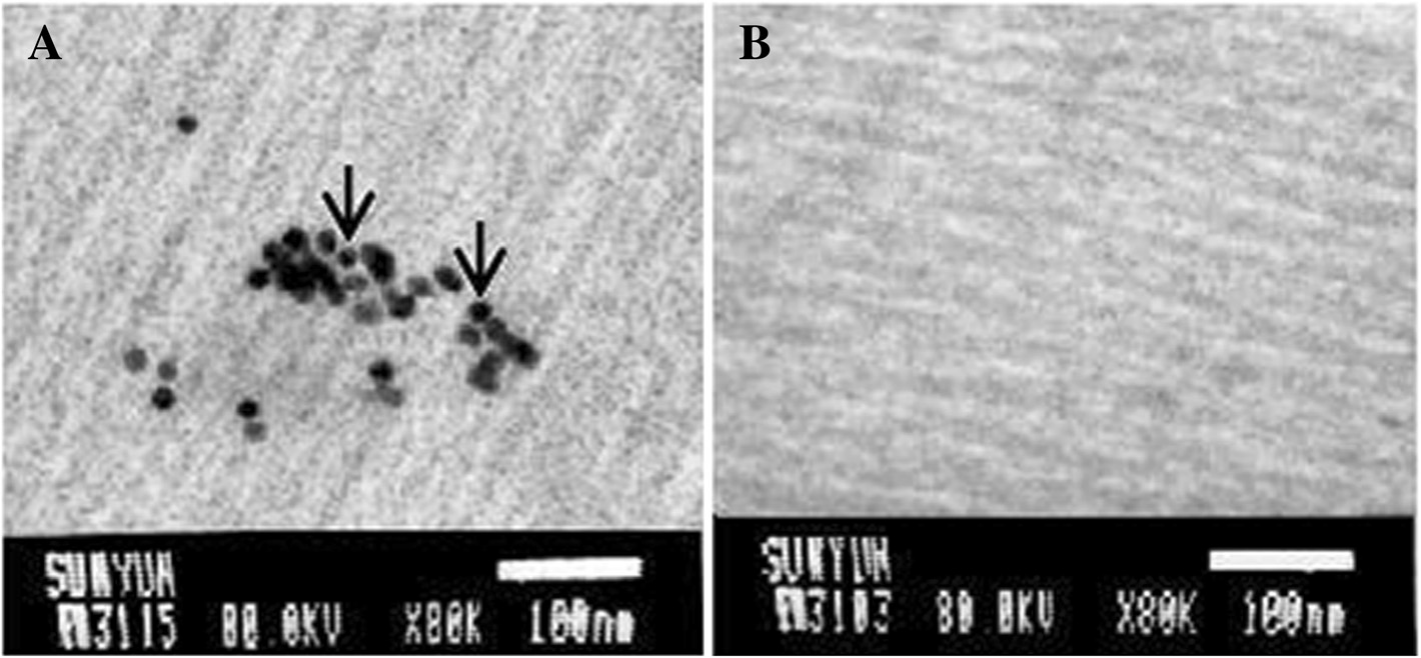
**Detection of SagE in vaccinated fish.** Muscle tissues were taken from flounder vaccinated with pSagE **(A)** and pCN3 **(B)** at 7 days post-vaccination and used for immunocolloidal gold electron microscopy with gold-labeled antibody. Arrows indicate gold particles. Bar = 100 nm.

A SagE-expressing DNA vaccine, pSagE, was constructed. To examine the immunoprotective potential of pSagE, flounder were vaccinated with pSagE, the control vector pCN3, or PBS. At 7 days pv, PCR and RT-PCR were conducted to examine the presence of the vaccine plasmid and transcription of the vaccine gene respectively in muscle, kidney, liver, and spleen. The results showed that PCR detected pSagE and pCN3 in pSagE- and pCN3-vaccinated fish respectively but not in PBS-vaccinated fish, while RT-PCR detected *sagE* mRNA in pSagE-vaccinated fish only (Figure 1 and data not shown). To examine whether SagE protein was produced in the vaccinated fish, immunocolloidal gold electron microscopy was carried out, which detected SagE protein in the muscle tissues of pSagE-vaccinated fish but not in pCN3- or PBS-vaccinated fish (Figure 2 and data not shown).

### Protection induced by pSagE

The vaccinated fish were challenged at one and two months pv with *S. iniae* and monitored for mortality. The results showed that the accumulated mortalities of pSagE-, pCN3-, and PBS-vaccinated fish were 4%, 76%, and 82% respectively at one month pv, and 10%, 72%, and 80% respectively at two months pv. Hence, with PBS as a control, the RPS rates of pSagE-vaccinated fish were 95% and 88% at one and two months pv respectively.

### Immune response induced by pSagE

#### ***Expression of immune genes***

**Figure 3:**
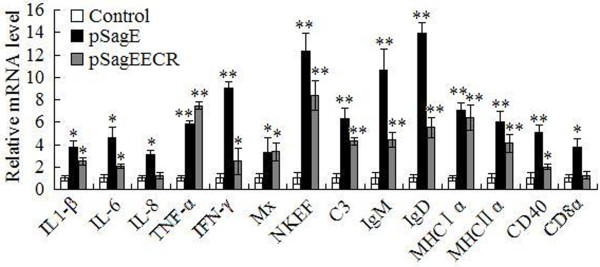
**Expression of immune genes in vaccinated fish.** Flounder were vaccinated with pSagE, pSagEECR, or pCN3 (control). Spleen was taken from the fish at 24 h post-challenge. Total RNA was extracted from the spleen and used for quantitative real time RT-PCR. For each gene, the mRNA level of the control fish was set as 1. Data are presented as means ± SE (N = 3). ^*^P < 0.05; ^**^P < 0.01.

To investigate whether pSagE vaccination affected gene expression, qRT-PCR was conducted to examine the transcription level of immune genes in the spleen of pSagE- and pCN3-vaccinated fish at 24 h post-challenge. The immune genes examined were interleukin (IL)-1β, IL-6, IL-8, tumor necrosis factor alpha (TNF-α), interferon (IFN)-γ, interferon-induced Mx protein, natural killer enhancing factor (NKEF), complement C3, immunoglobulin M (IgM) and D (IgD), major histocompatibility complex (MHC) class Iα and class IIα, CD40, and CD8α. The results showed that compared to control fish, fish vaccinated with pSagE exhibited significantly enhanced expression of all examined genes, with relatively high levels of induction (more than 5-fold) observed with TNF-α, IFN-γ, NKEF, C3, IgM, IgD, MHC Iα, MHC IIα, and CD40 (Figure 3).

### Serum antibody production

**Figure 4:**
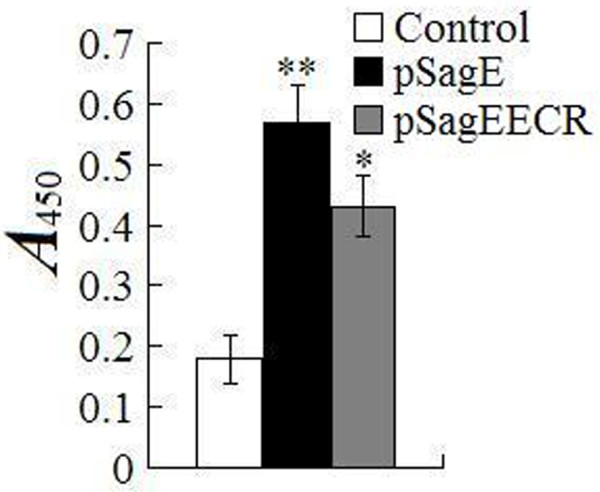
**Serum antibody production in vaccinated fish.** Sera were taken from flounder vaccinated with pSagE, pSagECR, and PBS (control). Serum antibodies against ECR were determined by ELISA. Values are shown as means ± SE (N = 5). ^*^P < 0.05; ^**^P < 0.01.

ELISA showed that at one month pv, fish vaccinated with pSagE produced serum antibodies that recognized recombinant ECR (rECR) (Figure 4). A comparable level of antibody production was detected in pSagE-vaccinated fish at two months pv. No serum antibodies against rECR were detected in pCN3-vaccinated fish.

### Interaction between serum antibodies and bacterial cells

**Figure 5:**
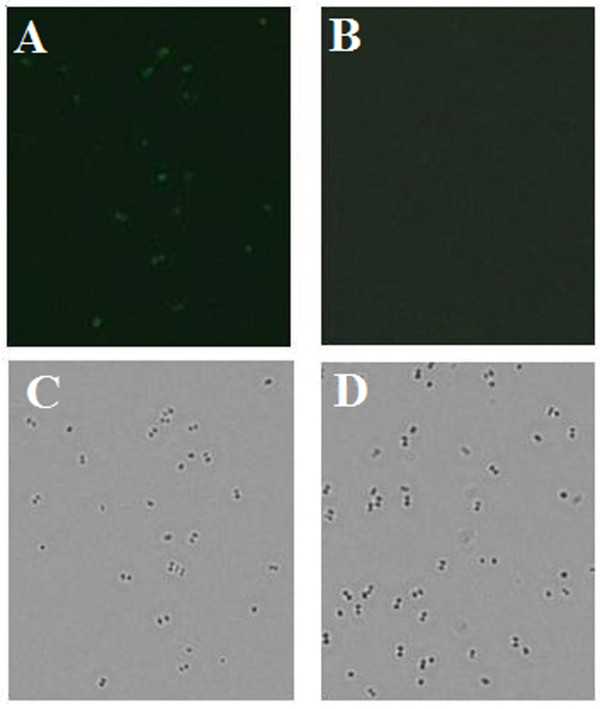
**Interaction between vaccine-induced antibodies and bacterial cells.***Streptococcus iniae* was incubated with serum from pSagE-vaccinated fish **(A and C)** or with serum from control fish **(B and D)**. Cell-bound antibodies were detected with FITC-labeled antibody. The cells were observed under a microscope with **(A and B)** or without **(C and D)** fluorescence.

Since SagE was predicted to be a membrane-localized protein, we examined whether it could be recognized in its natural state in *S. iniae* by pSagE-induced antibodies. For this purpose, live *S. iniae* was incubated with the serum from pSagE- or pCN3-vaccinated fish. The cell-bound antibodies were detected by FITC-labeled antibody. Microscopic examination showed that fluorescence was observed on the bacteria that had been treated with the serum from pSagE-vaccinated fish but not on the bacteria that had been treated with the serum from pCN3-vaccianted fish (Figure 5).

### Effect of pSagE-induced antibodies on bacterial infection

To further examine whether pSagE-induced antibodies had any effect on *S. iniae* infection, the bacteria were treated with the serum from pSagE- or pCN3-vaccianted fish before being used for infection of flounder FG cells. The number of bacterial cells that succeeded in infection was subsequently determined. The results showed that compared to treatment with pCN3-induced serum, treatment with pSagE-induced serum significantly reduced (6.4-fold lower) the number of *S. iniae* recovered from FG cells [see Additional file 3].

### Localization of the immunoprotective region of SagE

Since ECR is the major extracellular region of SagE, we examined its immunoprotective potential. For this purpose, flounder were vaccinated with pSagEECR, which expresses His-tagged ECR, pCN3, or PBS. At 7 days pv, RT-PCR detected the mRNA of ECR in the muscle, spleen, and kidney of pSagEECR-vaccinated fish, while immunocolloidal gold electron microscopy detected ECR protein in the muscle tissues of pSagEECR-vaccinated fish (data not shown). After challenging with *S. iniae* at one month pv, the accumulated mortalities of pSagEECR-, pCN3-, and PBS-vaccinated fish were 23%, 62% and 72% respectively. Hence, the RPS rate of pSagEECR was 68% with PBS as a control.

ELISA showed that fish vaccinated with pSagEECR produced specific serum antibodies, but the antibody level was significantly lower than that in fish vaccinated with pSagE (Figure 4). qRT-PCR analysis showed that pSagEECR-vaccinated fish exhibited significantly enhanced expression of IL-1β, IL-6, TNF-α, IFN-γ, Mx, NKEF, complement C3, IgM, IgD, MHC Iα, MHC IIα, and CD40 (Figure 3); however, the induction folds of IL-6, IFN-γ, IgM, and IgD were lower than those in pSagE-vaccinated fish.

## Discussion

It is known that in teleost, as in mammals, DNA vaccine plasmids administered via i.m. injection are transported to internal tissues, and the exogenous vaccine genes are expressed via the host’s expression system [39-41]. Likewise, in our study we found that following vaccination of flounder with pSagE, *sagE* mRNA and SagE protein were detected in multiple tissues at 7 days pv, suggesting that the DNA plasmid was taken up by host cells in local and distal tissues, where the *sagE* gene was successfully expressed. After challenge with *S. iniae* at one month pv, fish vaccinated with pSagE exhibited 95% survival, suggesting that an effective protective immunity was induced by pSagE in the vaccinated fish. At two months pv, pSagE-vaccinated fish displayed a RPS of 88%, which is comparable to that at one month pv, suggesting that the protective effect of pSagE lasted without significant decline for at least two months.

Compared to other types of vaccines, DNA vaccine possesses the advantage of being able to elicit systemic immune response of both the humoral and the cellular arms [42,43]. In the case of pSagE, qRT-PCR analysis showed that fish vaccinated with pSagE exhibited significantly enhanced expression of a range of genes, notably TNF-α, IFN-γ, NKEF, C3, IgM, IgD, MHC Iα, MHC IIα, and CD40, suggesting that in vivo expression of SagE stimulated innate and adaptive immunity. In line with the elevated expression of IgM, ELISA detected production of SagE specific serum antibodies in the vaccinated fish. Immunofluorescence microscopy showed that the antibodies from pSagE-vaccinated fish were able to bind live *S. iniae*, suggesting that SagE is naturally exposed on the cell surface of *S. iniae*.

Cellular infection analysis showed that when *S. iniae* was treated with pSagE-induced serum before incubation with FG cells, the number of bacteria recovered from the infected host cells was significantly reduced compared to that of the *S. iniae* pre-treated with the control serum. These results suggest that interaction between anti-SagE antibodies and the SagE on the surface of *S. iniae* inhibits *S. iniae* infection. Given the known involvement of streptolysin in *S. iniae* virulence [28,30], our results support the hypothesis that the immunoprotectivity of pSagE is probably due at least in part to its ability to induce production of specific serum antibodies, which, upon encountering *S. iniae* during subsequent infection, may inhibit the function of SagE and thus block *S. iniae* infection. Since ECR is the major extracellular region, we examined its potential protective effect. We found that the ECR-expressing DNA vaccine pSagEECR produced a RPS of 68%, suggesting that ECR contributes importantly to the protective effect of SagE. These results are consistent with those of ELISA analysis, which detected production of specific serum antibodies in pSagEECR-vaccinated fish, and qRT-PCR analysis, which showed that the expression patterns of immune genes induced by pSagEECR were largely similar to those induced by pSagE. However, both the antibody level and the magnitude of gene induction in pSagEECR-vaccinated fish were lower than those in pSagE-vaccinated fish, which may account for the lower protection of pSagEECR (compared to pSagE) and also suggest that in addition to ECR, there exit other immunogenic regions that are required to induce the full protective immunity observed with pSagE.

## Conclusion

In conclusion, we in this study developed a DNA vaccine, pSagE, which induces highly protective immunity against *S. iniae*. The protective effect of pSagE is probably due to its ability to elicit systemic immune response, in particular that of the humoral branch, which leads to production of specific serum antibodies that impair bacterial infection. These results not only provide an effective candidate vaccine against streptococcosis but also add insights to the immunoprotective mechanism of teleost DNA vaccine.

## Competing interests

The authors declare that they have no competing interests.

## Authors’ contributions

All authors participated in the study design. YS performed the experiments and acquired the data. LS designed the experiment. MX participated in the qRT-PCR analyses and the statistical analyses. CL participated in the animal experiment. YH made contributions to the conception of the study and wrote the manuscript. All authors revised the manuscript, and approved the final manuscript.

## Supplementary Material

Additional file 1 SDS-PAGE analysis of purified recombinant ECR (rECR). Purified rECR (Lane 2) was resolved by SDS-PAGE and viewed after staining with Coomassie brilliant blue R-250. Lane 1, protein markers. Click here for file

Additional file 2 Amino acid sequence of SagE. The putative signal peptide sequence is in italics, and the extracellular region ECR is underlined and in red. Click here for file

Additional file 3 Effect of vaccine-induced antibodies on bacterial infection. FG cells were infected with *Streptococcus iniae* that had been treated with serum from fish vaccinated with pSagE or pCN3. The number of bacteria recovered from the infected FG cells was determined by plate count. Data are presented as means ± SE (N = 4). ^**^P < 0.01. Click here for file
