# Supplementary figures and images for: SagE induces highly effective protective immunity against Streptococcus iniae mainly through an immunogenic domain in the extracellular region
Source: Acta Vet Scand. 2013 Nov 12;55(1):78. doi: 10.1186/1751-0147-55-78 (PMC3829104; doi:10.1186/1751-0147-55-78)

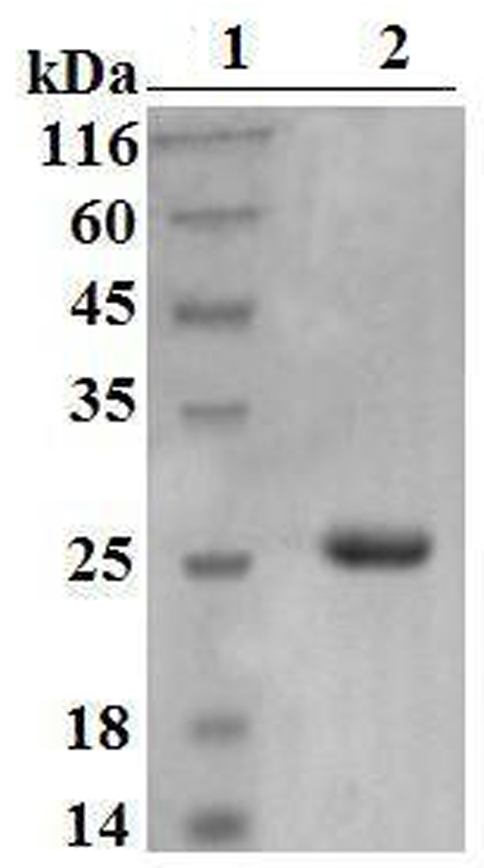

Supplement: Additional file 1 — SDS-PAGE analysis of purified recombinant ECR (rECR). Purified rECR (Lane 2) was resolved by SDS-PAGE and viewed after staining with Coomassie brilliant blue R-250. Lane 1, protein markers. [file 1751-0147-55-78-S1.tiff]

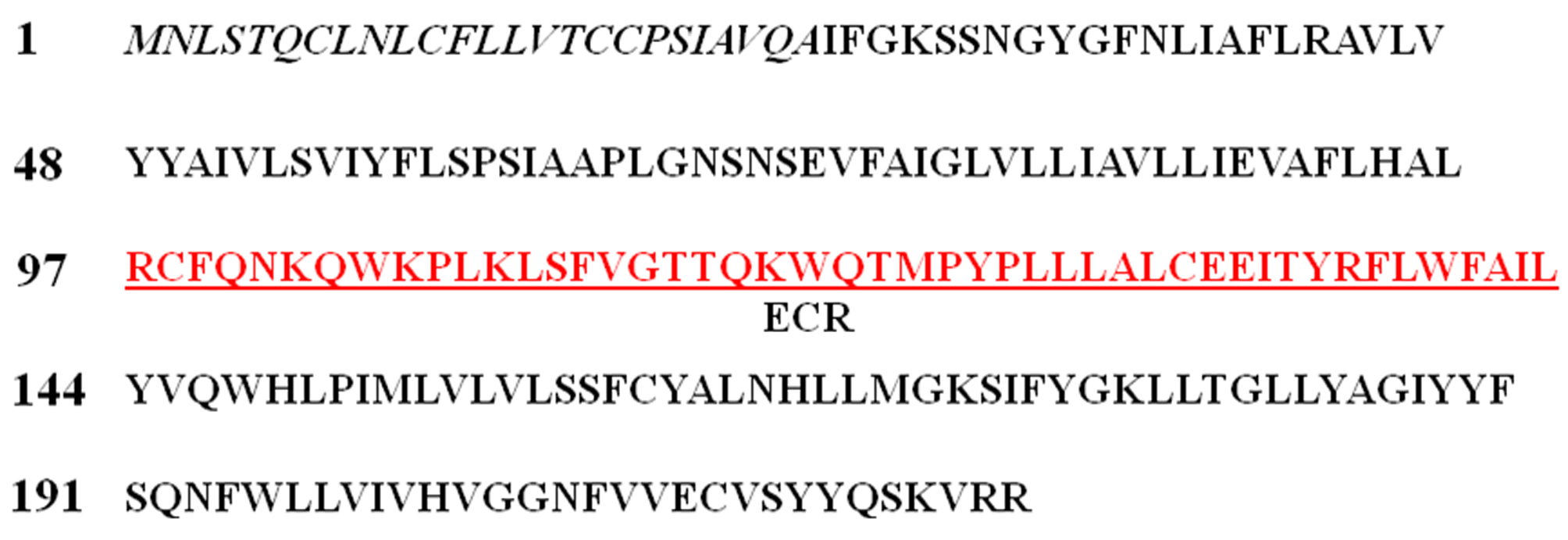

Supplement: Additional file 2 — Amino acid sequence of SagE. The putative signal peptide sequence is in italics, and the extracellular region ECR is underlined and in red. [file 1751-0147-55-78-S2.tiff]

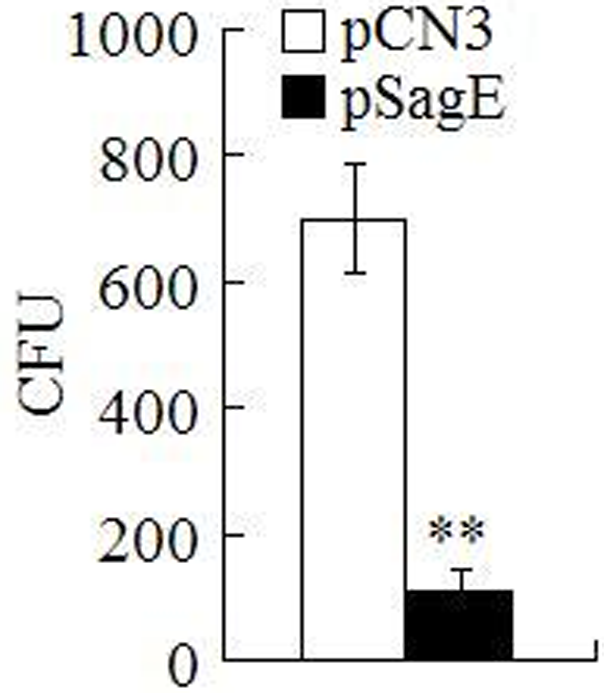

Supplement: Additional file 3 — Effect of vaccine-induced antibodies on bacterial infection. FG cells were infected with Streptococcus iniae that had been treated with serum from fish vaccinated with pSagE or pCN3. The number of bacteria recovered from the infected FG cells was determined by plate count. Data are presented as means ± SE (N = 4). **P < 0.01. [file 1751-0147-55-78-S3.tiff]
